# Genetic markers of stomatal cluster development in *Begoniaceae* revealed through trait analysis assisted by interactive deep-learning

**DOI:** 10.1093/plphys/kiag496

**Published:** 2026-07-13

**Authors:** Thu Phan Ly, Bryan M Williams, Abhijit Karnik, Catherine A Kidner, Rucha Karnik

**Affiliations:** Plant Science Group, School of Molecular Biosciences, College of Medical, Veterinary and Life Sciences, University of Glasgow, Glasgow G12 8QQ, United Kingdom; School of Computing and Communications, Lancaster University, Lancaster LA1 4WA, United Kingdom; School of Computing and Communications, Lancaster University, Lancaster LA1 4WA, United Kingdom; School of Biological Sciences, University of Edinburgh, Edinburgh EH9 3JW, United Kingdom; Royal Botanic Gardens Edinburgh, Edinburgh EH3 5LR, United Kingdom; Plant Science Group, School of Molecular Biosciences, College of Medical, Veterinary and Life Sciences, University of Glasgow, Glasgow G12 8QQ, United Kingdom

## Abstract

Stomata of plants track the immediate demand for carbon dioxide for photosynthesis while limiting transpirational water loss. Solitary stomatal patterns are common, yet some land plants develop noncontiguous stomatal clustering, where 2 or more stomata occur in groups and overlay a single air cavity. Clustering improves stomatal efficiency, reduces plant water use, and increases resilience to environment stress. How cluster development and physiology interact and integrate with the environment are open questions.

Here we used TESSERA, a deep-learning platform for stomatal detection with an interactive interface for data review. Tracking *Begonia* stomatal clustering patterns across various *Begonias*, we have uncovered correlations for stomatal clustering traits. The stomatal parameter data were applied to identify genetic loci involved in *Begonia* stomatal development using quantitative trait locus analysis. Combined with differential gene expression to refine the candidate list, our analysis reveals known and potential new *Begonia* candidates in stomatal development. As a test of this knowledge, we cloned *Begonia* SPEECHLESS (*Beg*SPCH), a loci identified in this screen and an established development-related gene in *Arabidopsis*. Unexpectedly, *Arabidopsis spch-3* mutants transformed to express *Beg*SPCH developed stomatal clusters unlike the mutant plants expressing *At*SPCH. Thus, various molecular and environmental factors likely overlay transcriptional regulation in stomatal development.

## Teaser

Stomatal parameter data for *Begonias* using a deep-learning platform reveal correlations for stomatal cluster-configurations. Combined quantitative trait locus and gene expression analysis reveals potential targets for stomatal development providing new opportunities for crop engineering.

## Introduction

Stomata are tiny pores generally occurring of the leaf surface that control the diffusion of atmospheric carbon dioxide (CO_2_) into the leaf for photosynthesis in exchange for transpirational water loss (Lawson and Blatt 2014). Acting as guardians, stomata limit entry of microbial pathogens into the plant (Melotto et al. 2006) and contribute to the regulation of internal leaf temperature (Lawson and Vialet-Chabrand 2019). Thereby the efficiency with which stomata respond to changes in the environment underpins plant health and agricultural productivity. Indeed, stomata are crucial yet less utilized targets to improve crop efficiency.

Stomata open or close within minutes in response to signals, notably light, humidity, CO_2_, and pathogens and in water stress or abscisic acid (ABA) (Melotto et al. 2006; Lawson and Blatt 2014; Baena et al. 2022). The regulation of stomatal movements is governed by dynamic, turgor-driven changes in the volume of the pair of specialized guard cells that surround the stomatal pore (Jezek and Blatt 2017). A large body of research data on stomata relates to transpiration and carbon assimilation because of kinetics of stomatal movements; see reviews (Jezek and Blatt 2017; Nguyen et al. 2024). Past work suggests that mutations that alter stomatal kinetics to increase water use efficiency commonly do so at the expense of carbon assimilation, reflecting a trade-off between CO_2_ availability within the leaf and transpiration (Lawson and Blatt 2014; Jezek and Blatt 2017; Lawson and Vialet-Chabrand 2019).

Stomatal morphology and patterning on the leaf epidermis significantly contribute to the control of stomatal movements in relation to its surrounding cells (Peterson et al. 2010; Chater et al. 2017). Thereby developmental changes, including stomata shape, size, and arrangement, underpin stomatal behaviors in ambient conditions and under environment stress (Doheny-Adams et al. 2012; Chater et al. 2017; Liu et al. 2023). These and other micromorphological stomatal traits are sensitive to environmental and endogenous cues. In paleoecology, for example, parameters such as stomatal index, defined as the ratio between the number of stomata and epidermal cells of fossil plant cuticles, can provide insights into the atmospheric CO_2_ levels in a given era (Beerling and Chaloner 1993). Indeed, CO_2_ influences dynamic stomatal movements and the coordinate development of stomatal patterns in the long term (Casson and Gray 2008). For instance, elevated CO_2_ signals promote immediate closing of the stomatal pore, and in the long term it suppresses biogenesis, affecting stomatal density and size in developing leaves (Lake and Woodward 2008; Gan et al. 2010).

In dicotyledonous plants, a pair of kidney-shaped guard cells surround the stomatal pore, while in grass species such as barely and maize, each stomatal pore is lined by dumbbell-shaped guard cells surrounded by a pair of auxiliary subsidiary cells (Cai et al. 2017). Stomatal development follows a series of asymmetric and symmetric divisions of protodermal cells to form guard cells driven by a cascade of regulated processes and transcription factors (see review, Zoulias et al. 2018). In most land plants analyzed to date, including the popular plant model *Arabidopsis thaliana*, stomatal development follows a 1-cell spacing rule, where each stoma has 1 smaller and 2 larger neighboring cells (Zhao and Sack 1999; Berger and Altmann 2000; Gray 2007) that prevent direct contact between stomata (Gray 2007; Chater et al. 2017).

Stomatal clusters develop as an adaptation to environment stress, where 2 or more stomata overlay a single air cavity (Papanatsiou et al. 2017; Tsai et al. 2022). Stomatal clustering is induced in *Arabidopsis* with the loss of TOO MANY MOUTH (TMM) gene function. Stomatal clusters in *tmm1* mutants are contiguous where the epidermal cells are only observed between the clusters but never interspaced between stomata within a cluster. Past studies show that stomatal dynamics are impaired in *tmm1* mutants together with reduced accumulation of K^+^ in guard cells (Papanatsiou et al. 2016), suggesting that spacing between stomata is important to ensure proper stomatal behavior. Several *Begonia* species have naturally formed noncontiguous stomatal clusters with subsidiary cells that prevent direct contact between adjacent stomata in the cluster (Boghdan and Barkley 1972; Hoover 1986; Tsai et al. 2022). The development of stomatal clusters is linked to environment sensing, although underpinning molecular mechanisms that act in noncontiguous stomatal cluster development are unknown.

Past studies suggest that exposure to drought and high salt drives stomatal clustering in *Vicia faba* (broad bean) (Gan et al. 2010). In turn, clustering influences stomatal physiology. For example, in *Begonias*, clustering enhances stomatal performance and plant water use efficiency (WUE) compared with solitary stomata under saturated light (Papanatsiou et al. 2017; Tsai et al. 2022) and can promote growth in a challenging environment (Rudall et al. 2018). Fundamentally, how stomatal development and physiology connect are open questions.

Here we trained a deep-learning–based model for stomata detection integrating a web-based interactive user interface for data review and amenable for analysis of solitary and clustered stomata parameters with speed and precision. Using low-resolution scans of the leaf epidermis imprints as input, machine-generated stomatal parameter data were used for building correlations for stomatal cluster patterning traits in mapping populations of *B. conchifolia* and *B. plebeja* species with stomatal clustering. Quantitative trait locus (QTL) analysis using phenotype and genetic information was combined with differential gene expression data to reveal with high confidence a subset of multifunctional genes that likely influenced stomatal cluster development. Remarkably, the expression of a *Begonia* transcription factor gene that drives solitary stomata biogenesis transferred the stomatal clustering trait into *Arabidopsis*. With the crisis for global climate change threatening agricultural food production and freshwater availability, this new knowledge is valuable for fundamental stomatal biology research and the engineering of climate resilient crops.

## Results

### Stomatal patterning on leaf epidermis of plants from the *Begoniaceae* genus indicates diverse stomatal clusters

**Figure 1:**
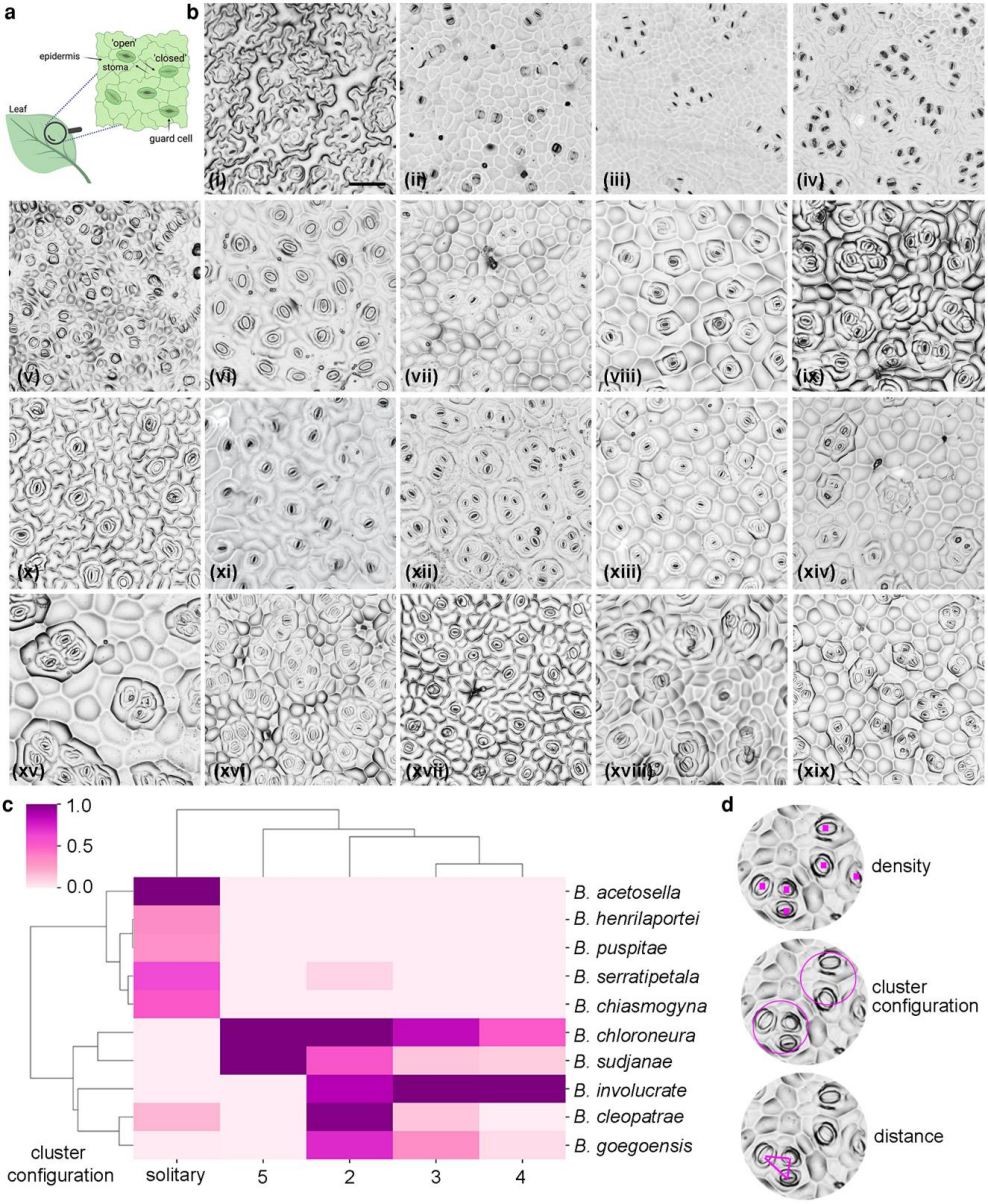
Diversity in stomatal patterns and their analysis parameters. **a)** Schematic showing typical positioning of stomata on abaxial leaf surface. Stomata with open and closed pores are depicted. Schematic created in BioRender. **b)** Representative images of leaf epidermis acquired using imprints (see Fig. S1). A diverse set of stomatal patterns including stomata in clusters are shown from: (i) *Arabidopsis thaliana*, and *Begonia* species (ii) *B. conchifolia*, (iii) *B. foliosa*, (iv) *B. odorata*, (v) *B. plebeja*, (vi) *B. serratipetala*, (vii) *B. involucrate*, (viii) *B. henrilaportei*, (ix) *B. corredorana*, (x) *B. puspitae*, (xi) *B.ulmifolia*, (xii) *B. subciliata*, (xiii) *B. chiasmogyna*, (xiv) *B. goegoensis*, (xv) *B. glandulifera*, (xvi) *B. chloroneura*, (xvii) *B. acetosella*, (xviii), *B. cleopatrae*, (xix) *B. sudjanae*. Scale bar = 200 μm. **c)** Heatmap depicting hierarchical clustering of the mean for measurements of solitary stomata density cluster-size configurations in 10 representative *Begonia* species. Data are *n* = 3 images each. **d)** Schematic representation of stomatal trait parameters identified for phenotype analysis: stomatal density (top panel), cluster configuration (middle panel), and distance between adjacent stomata in a cluster (bottom panel).

Stomata normally occur on the abaxial leaf surface (Fig. 1a). Past studies have reported numbers, size, and shape of stomata using the analysis of microscope images of leaf epidermal peels or epidermis imprints. Distinct from the canonical solitary stomata, noncontiguous stomatal clusters are reported in the *Begonia* genus, as well as in other taxa such as *Rubiaceae*, *Saxifragaceae*, and *Gesneriaceae* (Hoover 1986; Van Cotthem 2008). To track stomatal patterns, we chose as representatives of the mega–diverse *Begoniacea*e genus (Moonlight et al. 2018) plants from 20 different *Begonia* species housed at the Royal Botanical Gardens Edinburgh (UK).

Epidermal peels or fluorescence imaging of epidermis requiring advanced imaging equipment are commonly used for analysis of stomatal patterns, size, and aperture (Eisele et al. 2016; Millstead et al. 2020). However, characterizing stomatal patterns in a manner that is nondestuctive to the leaf can be challenging because leaf trichomes (hair) (Handley et al. 2005), dirt, and air bubbles can interfere with sample preparation and reduce image quality. Here, we adapted a previously reported protocol (Wacker et al. 2025) for preparing epidermal imprints to include steps for clearing the leaf surface of debri and trichomes prior to preparation of imprints. Positive impressions of leaf epidermis imprints were transferred to glass slides, and images were acquired at 20× with a slide scanner (see Methods, Fig. S1). Leaf epidermis imprint images acquired from several different *Begonia* species showed diversity in stomatal patterns, including solitary stoma and stomatal clusters with 2 or more stoma each (Fig. 1b).

We randomly chose a subset of 10 *Begonia* species for analysis and found significant diversity in stomatal patterns on leaf epidermis, with occurance of both solitary or clustered stomata as well as more than 1 kind of cluster configurations in a single species. To determine if stomatal cluster configurations were correlated, hierachically clustered linkage heatmap was generated (Fig. 1c). The approach (see Methods; Waskom 2021) is a prevailing visualization tool for revealing patterns and relationships within complex datasets. Our analysis suggested that solitary stomata are less likely to occur where clusters of 3 or more stoma are predominant. Where clusters of 4 or more stomata were formed, clusters with 2 or 3 stomata were also present, although cluster configurations with 2 or 3 stoma per cluster showed less variability in cluster configurations (Fig. 1c). Based on these stomatal patterns, we identified as parameters for phenotype analysis (Fig. 1d) the measurement of stomatal density, that is the stomatal count per unit epidermal surface stomatal cluster configuration that is the number of stoma in each cluster, and stomatal distance measured as the length across centers of 2 adjacent stomata in a cluster.

### Analysis of stomatal patterning with TESSERA, a human-computer interactive deep-learning model for tracking solitary and clustered stomata

**Figure 2:**
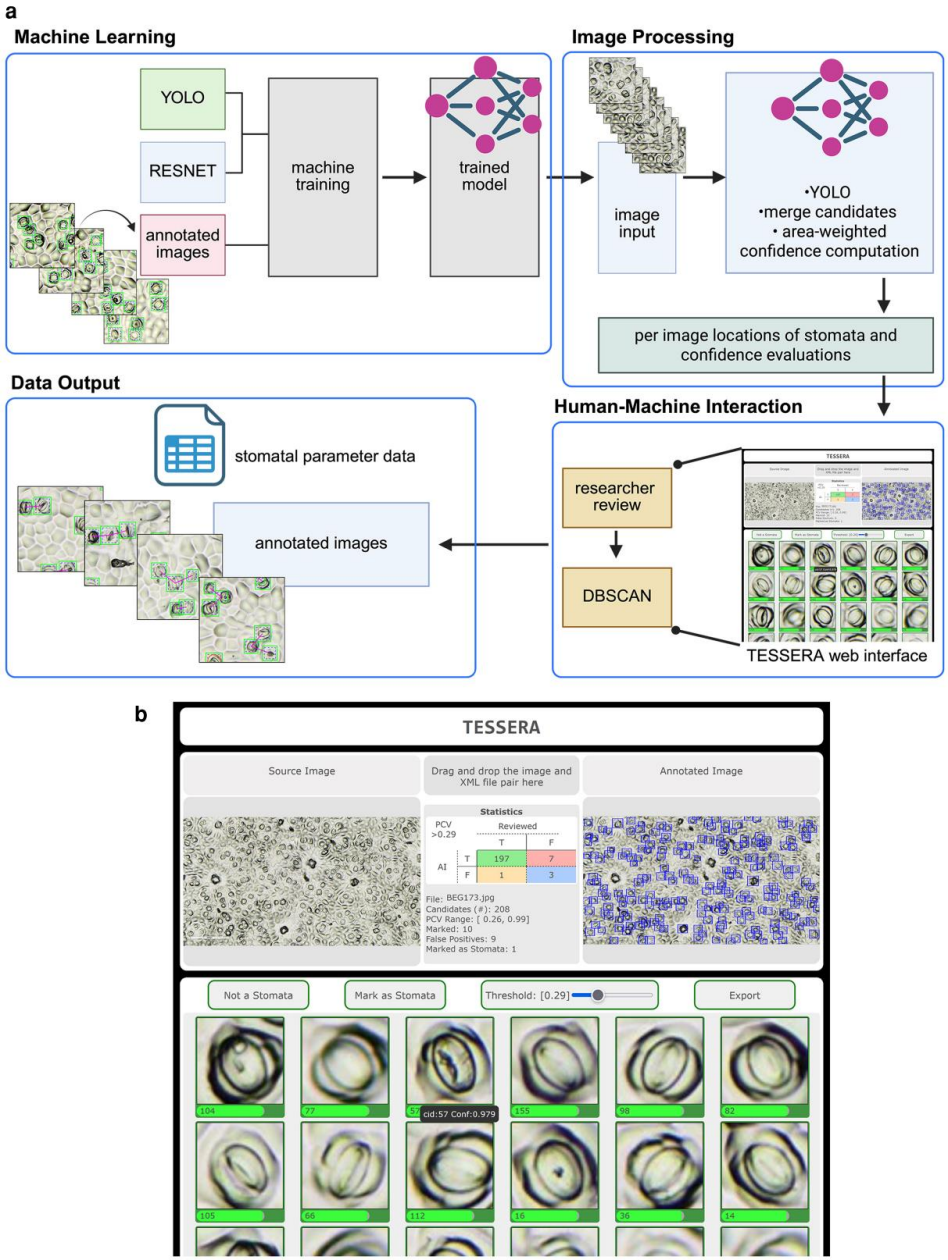
TESSERA pipeline. **a)** Schematic representing TESSERA pipeline, for machine learning, image processing, human-machine interactions, and data output steps (also see Methods). **b)** Screengrab of TESSERA machine-human web interface, showing input representative image (source image, top left), machine-annotated image (annotated image, top right), gallery of machine-detected stomata artefacts in the input image file (bottom), and live statistical analysis of machine performance displayed before and after interactive human review (statistics, top middle). Interactive interface (gallery, top panel) allows users to re-categorize selected artefacts in the gallery and by clicking “not a stomata” or “mark as stomata,” adjust the threshold value for detection to change stringency (threshold, gallery, top panel) and finally export parameter data (export, gallery, top panel). Schematic created in BioRender.

To achieve reliable and high-throughput stomatal pattern analysis, we turned to the use of machine-based deep-learning. We trained a deep-learning model for Topological Estimation of Stomatal Structures for Relational Modeling and Analysis (TESSERA) to track stomata occurring in clusters as well as solitary stomata (Fig. 2a; also see Methods). The “Machine Learning” module used as input representative images from 2 different *Begonia* species, the *B. conchifolia* (Fig. 1b viii) with solitary and 2 to 3 stomata clusters, and *B. plebeja* (Fig. 1b xi) with clusters of up to 4 to 5 stomata each (Papanatsiou et al. 2017; Tsai et al. 2022). Training images were annotated by an expert researcher designated as the “human trainer.” For machine training and image processing (Fig. 2a, “machine learning” module), we used YOLO version7 (Redmon et al. 2016; Joseph and Ali 2018) and RESNET (He et al. 2016), much as in the past with single stomata detection tools (Casado-García et al. 2020). TESSERA is designed to include additional outputs for trait analysis, including stomatal cluster configuration, stomatal density in clusters, and distance between stomata. As input for TESSERA, low-resolution scanned images of leaf epidermis imprints were uploaded for machine processing (Fig. 2a, “image processing” module). For integrating machine learning with human review, we designed TESSERA to include an interactive web interface (Fig. 2a, “human-machine interaction” module, Fig. 2b). Unlike in other similar platforms for stomatal analysis (Fetter et al. 2019; Casado-García et al. 2020), the web interface allows the users to visualize and review the machine-analyzed stomata artefacts and refine the output data selection through bulk filtering or single stomata inclusion and rejection. Following the user review, parameter data for solitarily and clustered stomata configurations computed by TESSERA together with machine-annotated images files are output for further processing and validation by the researchers (Fig. 2a, “data output” module). Thus, TESSERA supports deep-learning–based image data analysis with an interactive interface for review of output data that integrates advanced user control.

We tested the sensitivity of automated image processing by TESSERA in tracking stomata. Images of leaf imprints collected from 20 *Begonia* species with diverse stomatal patterns were used as input (cf. Fig. 1b for examples). The machine-generated data, prior to human review, was evaluated against the human trainer as the gold standard. For the analysis, human trainer data was derived using the review of the machine-generated data by using TESSERA web interface in addition to any stomata entirely missed by the machine. We calculated %machine sensitivity, which is the percentage of recall, the accurately predicted positive events out of all actual positive events, to be 89.80% and the F1-score that represents the precision and recall of a classification model to be 0.916 (see Methods and Fig. S2). At the time of implementation of TESSERA, we used YOLOv7 as our pattern detection model. Newer model variants are since available, and evidence suggests incremental gains in performance across YOLO v7 to v13 (Hakim et al. 2026). Our data demonstrate that TESSERA reliably and accurately identifies stomata in images from a range of *Begonia* species, including stoma occurring in both solitary and clustered patterns. As newer object detection models become available, incremental changes in model reliability and speed of pattern recognition can be expected.

**Figure 3:**
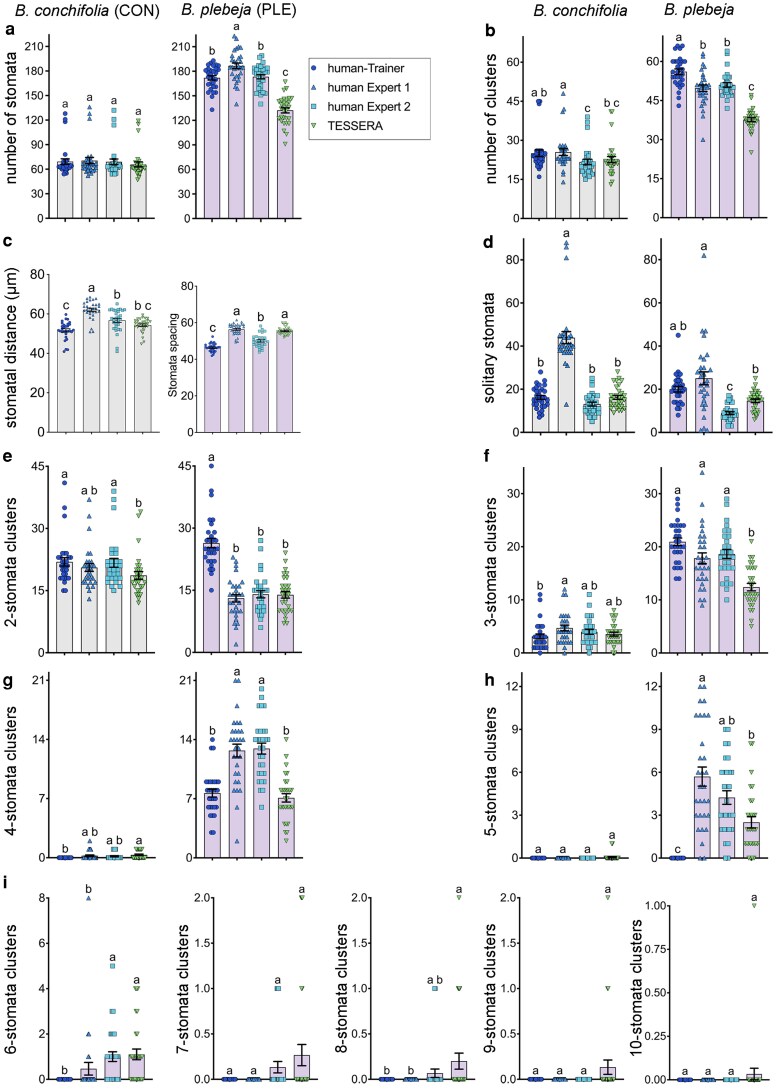
Comparing TESSERA performance with human data output. Graphs for mean data ± SE for parameter measurements obtained from TESSERA (inverted triangle), human trainer (circle), human expert 1 (triangle), and human expert 2 (square). **a-i)** Images from *B. conchifolia* and *B. plebeja* species were analyzed for number of stomata **(a)**, number of clusters **(b)**, distance between adjacent stomata in a cluster **(c)**, number of solitary stomata **(d)**, number of 2-stomata clusters **(e)**, number of 3-stomata clusters **(f)**, number of 4-stomata clusters **(g)**, number of 5-stomata clusters **(h)**, and number of 6-, 7-, 8-, 9-, or 10-stomata clusters **(i)**. The human researchers identified stomata visually and recorded measurements using ImageJ (Fiji). Data are *n* = 30 images for each species. Statistical significance is indicated using letters, determined using Brown–Forsythe and Welch ANOVA and Kruskal-Wallis tests. (*P* < 0.05).

Epidermis imprint images from *B. conchifolia* (CON) and *B. plebeja* (PLE) species as models were used to train TESSERA for analysis of different phenotypic traits associated with stomatal clustering, including total number of stomata, the number of clusters, number of different cluster configurations (eg clusters of 2- stomata or 3- stomata), and the distance between stoma in a cluster. To evaluate TESSERA training, Lin concordance corellation coefficient (CCC) and oridnary least square (OLS) analysis (see Methods) were used to compare analyzed data output from TESSERA and the human trainer (Fig. S3). These analysis suggested that TESSERA efficiently identified stomata in both CON and PLE, although the machine was more efficient in analysis of data from CON compared with the PLE species and that there were very few instances where clusters of 6 stomata or more were detected, although they did not exist (Fig. S3 h–j). We evaluated TESSERA performance in comparison with human researchers using mean parameter data outputs by the 2 researchers referred to as human “expert 1” and “expert 2” and as gold standard, the human “trainer.” Data for stomatal density and cluster parameters were evaluated and suggested that TESSERA output was comparable with the humans (Fig. 3). The human researchers all identified stomata visually and recorded measurements using ImageJ/Fiji (Schneider et al. 2012), a manual image data aquisition software. These data suggest that TESSERA detects stomata in solitary and clustered configurations with high accuracy and is able to track complex parameter trait data comparable with the human researcher.

### Analysis of stomatal patterns in *Begonias* suggest complex inter-relationships between phenotypic traits for clustered stomata

Using TESSERA to fast-track analysis, we characterized stomatal pattern phenotypes in CON and PLE parent lines and their mapping populations (see Methods). The species are phylogenetically close but not sister species (Moonlight et al. 2018), and they differ in stomatal cluster configurations, with CON exhibiting 2 to 3 stomata clusters and PLE with clusters of 4 or more stomata (Fig. 2). Mapping populations were generated by crossing CON and PLE plants to generate F1 progeny, which were backcrossed to each parent line to obtain mapping populations with genetic shuffling. The backcrossed populations of CON (CBC) and PLE (PBC) each exhibited combinations of clustered and nonclustered patterns (Figs. S4a and S5a). Performance tests for CBC (Fig. S4) and PBC (Fig. S5) suggest that TESSERA detects most stomatal traits with excellent accuracy (CCC > 0.8) compared with the human trainer.

**Figure 4:**
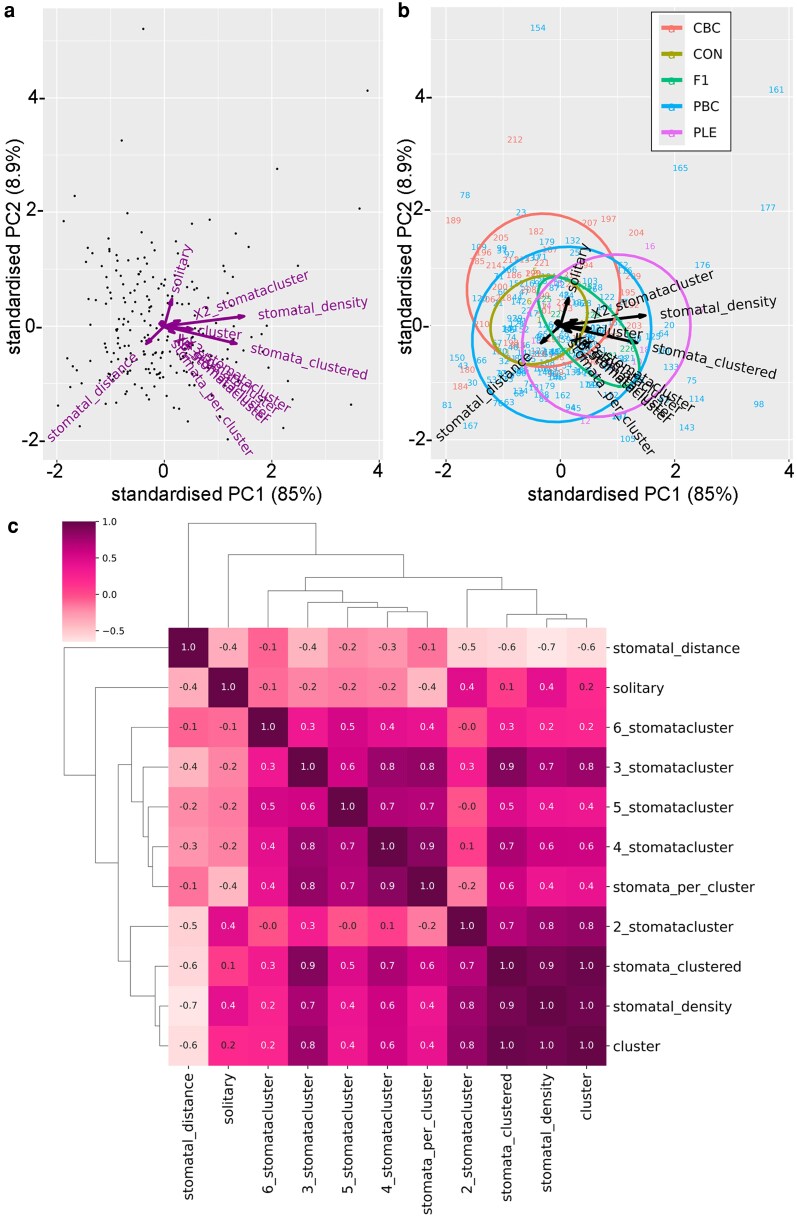
Stomatal patterning trait correlations derived from analysis of *B. conchifolia* and *B. plebeja* parent and backcrossed progeny. **a, b)** PCA with the cumulative proportions of variance for each of the first 2 PCs for 11 stomatal trait measurements (cf. Table 1). Analysis included plants from *B. conchifolia* [(CON), *n* = 10] and *B. plebeja* [(PLE), *n* = 10] parent plants and their progeny [(F1), *n* = 8], CON backcross [(CBC), *n* = 44] and PLE backcross [(PBC), *n* = 161] mapping populations. Scatter plot **(a)** present individual plants in *Begonia* mapping population with intermittent lines for the major loadings of variables to the axis. **b)** PCA analysis for variance distributions of 11 traits between the plant populations. Ellipse circle represents 95% confidence level distributions of each plant line populations. Number with distinctive color is labeled for each individual plant in each line (see inset). **c)** Pearson correlations of 11 stomatal traits within PBC backcross population. Hierarchically clustered heat map matrix presents the correlation number of each pair of 11 stomatal traits with −1 < r < 1: negative correlation, r = 0: no correlation, 0 < r < 1 for positive correlation.

Data from 11 different phenotypic traits for stomatal patterning (see Methods, Table S1) obtained from epidermis imprint analysis of every individual plant from the different lines were processed using principal component analysis (PCA) to resolve the relationships between the stomatal trait parameters (Fig. 4). PCA converts multi-dimensional phenotypic data for each trait into a small number of orthogonal principal components (PCs), which can be seen as trait combinations with similar characteristics, that cannot be measured directly and consolidates multiple traits and evaluates positive or negative correlations between each parameter data in the trait network. Of the 11 principal components identified in the PCA for stomatal traits across all plant lines (*n* = 231 plants; see Methods), PC1 and PC2 consolidated the majority of the stomatal traits, with 94% of the total variation (Fig. 4a). The incidence of solitary stomata and stomata spacing was mainly captured by PC2 (8.9%), while most of the remaining traits were included in PC1 (85%). The data for PC absolute values (Table S2) suggested that traits for total stomatal density, the density of stomatal clusters, stomatal distance, and density of solitary stomata are likely to have greater impact on variance within the PCA. The highest influence on pattern variances in PC1 was contributed by traits for stomatal density (48.69%) and the density of stomatal clusters (39.68%). In PC2, the solitary stomata density trait mainly influenced pattern variances across the plant populations (48.39%), with stomatal distance (19.98%) and the density of stomata occurring in clusters (19.46%) accounting for most impact (Table S3). These data suggest that stomatal traits associated with stomatal densities and distances between adjacent stoma in a cluster have the most impact on pattern variances.

To determine how the variance for traits in the different back-crossed plant populations correlate to each of the parent lines, we generated PCA-biplot of different traits of individual plants for each of the plant populations (Fig. 4b). The distribution of variances between CON and PLE parents showed distinctions in traits, with PLE showing a higher dispersion compared with CON suggesting higher variability in stomatal traits. The distributions of variances for *B. plebeja* backcross plants (PBC) overlapped that of CON and a majority of the PLE. These observations suggest that PBC populations likely capture most of the stomatal trait variances compared with CBC. Thus, CON and PLE species have distinct stomatal pattern traits, with PBC mapping populations showing significant trait variance overlapping with the parent lines.

Correlation analysis between all the stomatal traits in PBC populations, depicted using a heat map (Fig. 4c), showed positive Pearson correlations (r) in traits for stomatal density, cluster density, and density of clustered stomata (r > 0.9), while negative correlations were observed for stomata distance and stomatal density (r < −0.7) or stomatal cluster density (r < −0.6). The average number of stomata per cluster dictated the positive trends for larger clusters [(3-stomata cluster, r = 0.8), (4-stomata cluster, r = 0.9)]. Thus, some of the phenotypic parameters can be grouped for mutual impacts on pattern variability. Taken together, the PCA suggests complex inter-relationships between traits that create distinctions for stomatal patterns linked to clustering.

### QTL analysis reveals involvement of several loci in *Begonia* stomatal cluster development

How is stomatal clustering regulated in *Begonias*? To address this question, we used single-quantitative trait locus (QTL) analysis, a statistical approach used to discover the genetic architecture underlying complex quantitative traits by linking the phenotypic data of trait measurements in PBC populations with genotypic data (Kearsey 1998; Lynch and Walsh 1998). QTL analysis was performed using the qtl2 package in R (Broman and Sen 2009) and plants from the PBC populations (*n* = 187). We used 16 different stomatal phenotype traits with TESSERA (see Methods) for the analysis. Data from 3 additional traits, for cluster length, cluster width, and spacing between clusters were measured manually (Ali 2013). To track specific loci on *Begonia* chromosomes, the genetic map produced for PBC population (Brennan et al. 2012) was used in the analysis. Genotyping was improved with genome skims mapped to reference genome (Campos-Dominguez et al. 2025), and a new genetic map was produced by OneMap and analyzed by rQTL (Broman et al. 2003; Broman and Sen 2009).

**Figure 5:**
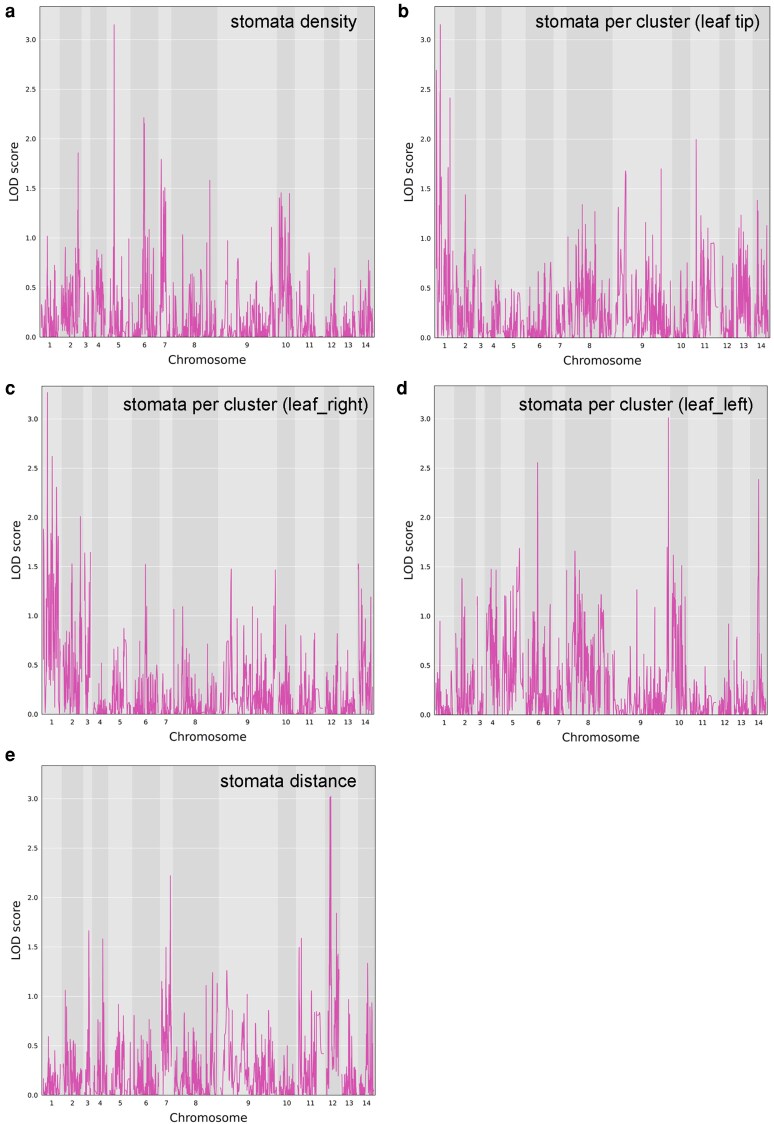
Analysis of stomatal clustering traits with significant QTLs. **a-e)** LOD plots (see Methods) for 5 significant QTLs at threshold above 3.0 and their peaks detected through 14 chromosomes of *B. conchifolia* from 5 phenotypic traits; (a) stomatal density, (b) average number of stomata per cluster measured from imprint images collected from leaf tip, (c) average number of stomata per cluster, leaf right region, (d) average number of stomata per cluster, leaf left region, and (e) distance (µm) between adjacent stomata inside clusters, leaf petiole region. Signal peaks detected from analysis across 14 linkage groups on chromosomes 1 to 14 of the *B. conchifolia* genome.

The genetic map for *Begonia conchifolia* has 14 linkage groups that range from ∼50 cM to ∼450 cM in length with 2,380 genetic markers. Both *B. plebeja* and *B. conchifolia* have 2*n* = 28 chromosomes (Campos-Domínguez et al. 2022), but the genome assembly is fragmented and correspondence between chromosomes and linkage groups is not possible without further sequencing and mapping. Mapping populations of under 2000 individuals and complex traits make detection of QTLs difficult (Liu et al. 2017), and none of our QTL had a logarithm of the odds (LOD) above 4.0. We used a stringent threshold of LOD 3.0 to pick 5 significant QTLs for 3 phenotypic traits located on linkage groups 1, 5, 9, and 12 (Fig. 5, Table 1).

**Table 1:** Significant QTL for stomatal traits.

Phenotype	Linkage group	Position	LOD	%var	Confidence interval (low)	Confidence interval (high)
Stomatal density	5	497.408	3.287	7.776	468.802	510.713
Stomata per cluster_leaf tip	1	329.804	3.181	7.535	13.804	1095.690
Stomata per cluster_leaf right	1	335.508	3.287	7.776	15.804	1199.862
Stomata per cluster—leaf left	9	4456.468	3.030	7.190	4338.819	4481.143
Stomatal distance_leaf petiole	12	283.047	3.123	7.403	245.619	802.590

Abbreviations: QTL, Quantitative Trait Loci; LOD, Logarithm of the Odds.

QTLs for the average number of stomata per cluster in different regions of leaf were identified on chromosomes 1 and 9 (Fig. 5b–d), accounting a total of 22.5% of the variations observed across the PBC population (Table 1). Stomata density was associated with chromosome 5, and a region regulating stomata spacing was detected on chromosome 12; both explained ∼7% of the variation in each trait. None of these traits co-localized with others. Scanning of the annotated genes on the 210 scaffolds underlying significant QTLs resulted in the identification of 908 potential gene candidates with assigned gene functions using *Arabidopsis* reference genome. These included transcription factor SPEECHLESS (SPCH), which directs the first asymmetric division initiating stomatal lineage (Pillitteri et al. 2007).

**Figure 6:**
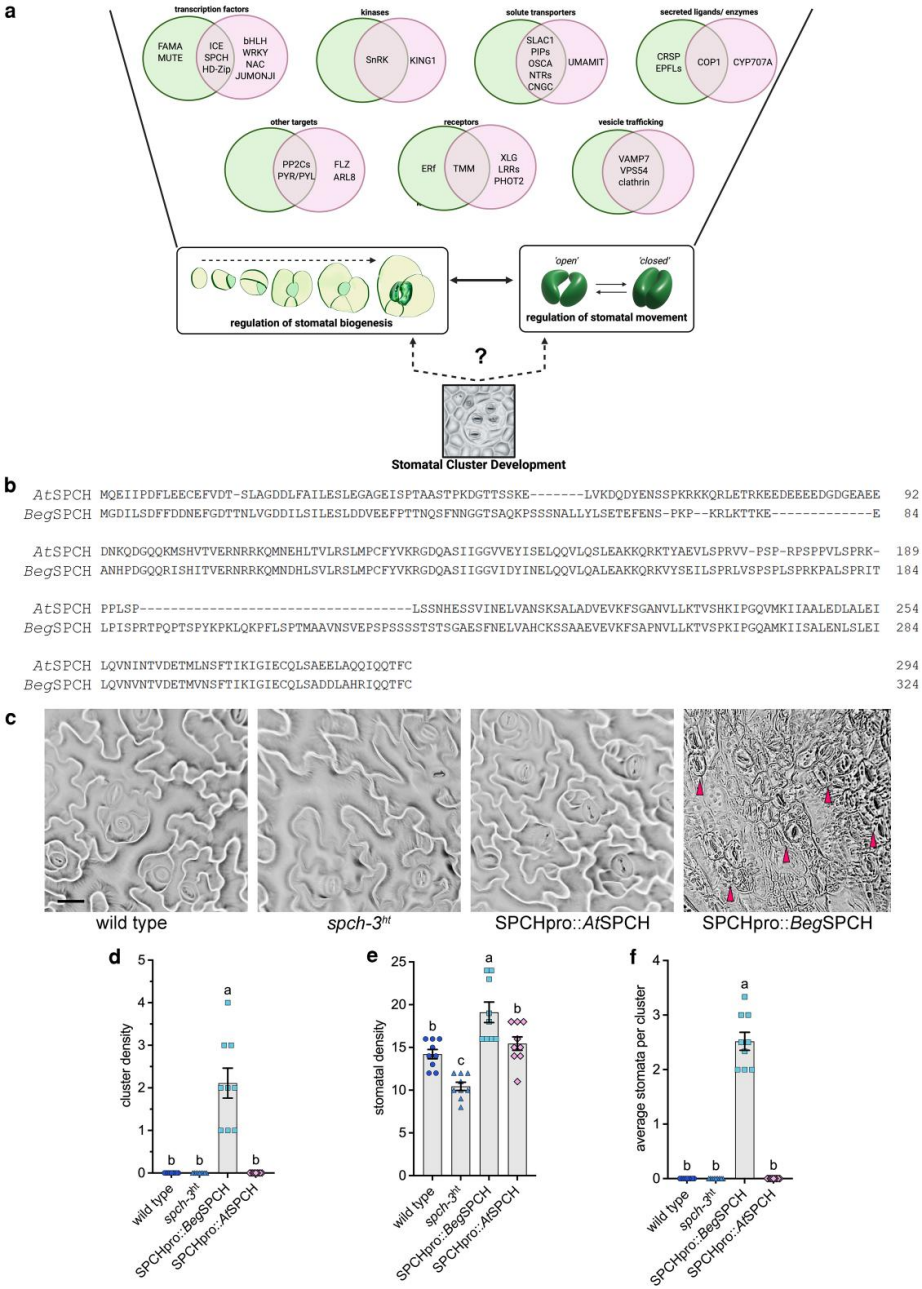
Schematic showing components identified in *Begonia* stomatal development and analysis of *Begonia* SPECHLESS. **a)** Gene/protein families in regulation of stomatal development and the control of stomatal movements are shown including (selected) candidates. Candidates linked to stomatal regulation based on existing knowledge in *Arabidopsis* (left circles) and potential new *Begonia* orthologs identified in this study (right circles) are shown. We suggest that noncontiguous stomatal clustering likely integrates pathways for stomatal biogenesis and stomatal movements. Schematic created in BioRender. **b)** Sequence alignment of *Arabidopsis* (*At*) and *Begonia* (*Beg*) SPCH protein sequences. **c)** Images, representative of *Arabidopsis* leaf epidermis imprints from wild type, *spch-3^ht^* (heterozygous) mutant, and complementation lines expressing GFP-tagged *At*SPCH and *Beg*SPCH under native *At*SPCH promoter. Red arrow marks stomatal clusters. Scale bar = 100 μm. **d-f)** Graphs for mean data ± SE in the plant lines showing per unit area, cluster density **(d)**, total stomatal density per unit area (nm^2^), **(e)** and average number of stomata per cluster **(f)**. Data are *n* = 9 images from 2 to 3 plant lines each. Statistical significance is indicated using letters, determined using Brown–Forsythe and Welch ANOVA and Kruskal-Wallis tests. (*P* < 0.05).

To further narrow the potential candidates, we used published RNASeq data (Emelianova et al. 2021) to examine differential expression analysis of these genes (DEGs) in leaf and vegetative bud tissues in *B. conchifolia* and *B. plebeja*. This analysis identified 138 candidate genes as more likely to act in Begonia stomatal development (Table S4, Fig. 6a). Included were genes for proteins implicated in stomatal responses to various environmental stimuli, including *Arabidopsis* orthologs of the so-called CONSTITUTIVE PHOTOMORPHOGENIC 1 (COP1) and PHOTOTROPIN PHOTORECEPTOR 2 (PHOT2) involved in light and temperature signaling (Fujii et al. 2017; Han et al. 2020), and EXTRA-LARGE G-PROTEIN 1 pathogen immunity (Wang et al. 2023); solute transporters including the guard cell anion transporter SLOWLY ACTIVATING ANION CHANNEL1 (SLAC1) and PLASMA MEMBRANE INTRINSIC PROTEIN (PIP1); proteins involved in vesicle traffic, such as VACUOLAR-PROTEIN-SORTING COMPLEX (VPS54) and VESICLE-ASSOCIATED MEMBRANE PROTEIN 7 isoforms (Bassham and Blatt 2008) (VAMP721); and protein modification enzymes such as SnRK1 KINASE complex and MITOGEN ACTIVATES PROTEIN KINASE4 (MAP4 K) implicated in stomatal biogenesis. There were 41 transcription factors identified, of which 9 are orthologs of the MYC-LIKE BASIC HELIX-LOOP-HELIX (bHLH) family implicated in plant responses to cold stress and stomatal development (MacAlister and Bergmann 2011), including INDUCER OF CBF EXPRESSION1 (ICE, SCREAM, SCRM1). Orthologs of the AUXIN RESPONSE FACTOR (ARF) family are important for auxin regulated plant growth and development (Yu et al. 2022), and epigenetic regulators JUMONJI family proteins associated with a variety of cellular processes likely contribute to stomatal development and clustering.

We also tested if the power of the QTL analysis could be further improved using as co-variants in the analysis a range of leaf anatomy and physiology traits previously measured by Ali (2013) (Table S1; also see Methods). These and other studies suggest that noncontiguous stomata clustering in *Begonia* might be influenced by leaf anatomy and physiology (Tang et al. 2002; Ali 2013; Rudall et al. 2018; Tsai et al. 2022). The linkage groups and positions for each covariate QTL showed no significant differences from the noncovariate analysis (Table S5). Two additional QTLs were identified for linkage groups 11 and 13 with the covariate–specific leaf area (SLA) trait (c.f Table 2), including a paralog of SPEECHLESS and 110 additional gene candidates with differential gene expression (Table S6).

**Table 2:** Significant QTL for stomatal traits with covariate SLA.

Phenotype	Linkage group	Position	LOD	%var	Confidence interval (low)	Confidence interval (high)
Cluster length	5	576.952	3.172	7.514	539.983	604.054
Clustered_leaf left	9	3245.078	3.463	8.175	3226.511	3283.879
Clustered_leaf tip	11	726.000	3.023	7.174	698.259	2068.328
	13	171.000	3.091	7.329	154.565	1039.543
2 stomata cluster_leaf petiole	12	330.427	3.446	8.137	224.751	997.542

Abbreviations: QTL, Quantitaitive Trait Loci; SLA, Specific Leaf Area; LOD, Logarithm of the Odds.

### Analysis of candidate *Begonia* gene identified in QTL using TESSERA

The combined QTL, gene expression, and sequence diversity across *Arabidopsis* and *Begonia* singled out SPEECHLESS as the most relevant for further verification of the findings using physiological analysis with transgenics. This is because it is well established that *Arabidopsis* SPCH is a key determinant of single stomatal organization (Lampard et al. 2008). Yet it was unexpected that *Beg*SPCH should appear as a factor determining stomatal clustering. We cloned *Begonia SPCH* (see Methods) and prepared transgenic lines expressing the *Beg*SPCH gene in *Arabidopsis spch-3^ht^* mutant background using floral dipping (Clough and Bent 1998; Xia et al. 2019). *Begonia SPCH* shares high sequence homology with its *Arabidopsis* homolog but also has distinct domains (Fig. 6b). We found that transgenic plants expressing *Beg*SPCH (SPCHp::*Beg*SPCH), but not the *At*SPCH (SPCHp::*At*SPCH), develop stomatal clusters (Fig. 6c and d). The SPCHp::*Beg*SPCH lines had a significantly higher number of stomata compared with the wild-type and SPCHp::*At*SPCH plants (Fig. 6e), with an average of 2 to 3 stomata per cluster. Taken together, these data show that new knowledge from studies in *Begonias* has the potential to inform future studies on plant development and stomatal engineering.

## Discussion

Stomata occur as solitary in most species of plants studied to date, including in *Arabidopsis thaliana*, the popular model for stomatal biology research. Unlike *Arabidopsis*, the *Begonia* species have naturally evolved to present noncontiguous stomatal clusters that share the same substomatal cavity (Ali 2013). Contiguous stomatal clusters can form in *Arabidopsis* due to loss of TMM function, but direct contact between adjacent guard cells influences dynamic stomatal movements (Papanatsiou et al. 2017). Nevertheless, stomatal clustering enhances WUE in *Begonias* and in *Arabidopsis tmm* mutants compared with wild-type plants (Dow et al. 2017; Tsai et al. 2022) due to the synchronization of responses and rapid stomatal kinetics (Papanatsiou et al. 2017). The vast *Begoniaceae* genus therefore presents opportunities for analysis of stomatal development with associated phenotypic traits attributed to changes in their habitats and environment stress (Chater et al. 2017). Our study with *Begonias* uncovers a set of physiologically related genes in plant environment sensing along with transcription factors in developmental patterning act together in stomatal development such as the *Arabidopsis* transcription factor SPCH, which initiates solitary stomata development. Interestingly, the expression of *Begonia* SPCH transcription factor in *Arabidopsis* drives stomatal clustering. Thus, our work in *Begonias* highlights the power of TESSERA in physiological parameter analysis and its relevance in the development of new knowledge for stomatal engineering.

### Deep-learning models integrating human-computer interaction strategies fast track research in stomatal biology

Studies with *Begonias* can be challenging as the plants may be slow growing and require particular horticultural care. Several of the species have significantly larger leaf surface area compared with *Arabidopsis*, requiring collection of more data points to map epidermal patterning. *Begonia* plants have diverse leaf structures and sizes and can have a higher number of trichomes on leaf surface, resulting in low-quality imprint images (Ali 2013). Stomatal clustering morphology and traits in *Begonia* are sparingly understood, with studies being limited by the lack of technologies for high-throughput analysis.

To study stomatal patterning, manual analysis of images by researchers is routine. For instance, the analysis of epidermal peels or epidermis imprints with an image processing software such as ImageJ (Schneider et al. 2012) using a computer interface to measure stomatal density or aperture size is common. However, such methods are laborious and time intensive and often require manual input from a skilled experimenter to yield reliable data (Gibbs and Burgess 2024). Moreover, manual analysis can have limitations for accuracy due to variabilities in human performance (Fig. 3). Instead, deep-learning tools offer significant savings for time (Fetter et al. 2019; Casado-García et al. 2020; Gibbs and Burgess 2024; Wacker et al. 2025) as they can rapidly generate large quantities of parameter data by offering fast and efficient object detection for analysis of complex biological images (Casado-García et al. 2020; Arya et al. 2022; Gibbs and Burgess 2024; Thai et al. 2025). Examples are the existing deep-learning algorithms using Convolutional Neural Networks (CNNs) such as Region-based Convolutional Neural Networks (RCNN) and You Only Look Once (YOLO), which we used for TESSERA, and/or the classification of objects based on individual pixel such as Mask R-CNN and U-Net (Bheemanahalli et al. 2021; Jayakody et al. 2021; Zhang et al. 2021). It can be expected that newer algorithms as they arise in the future could provide further performance gains through efficient processing, faster training, and reduced detection time, although no single model universally outperforms the others across all performance and deployment criteria as demonstrated in the analysis of YOLOv7 to YOLOv13 [see (Hakim et al. 2026)]. TESSERA allows fast-track stomatal pattern detection to be complemented by human intervention, thus improving on the F1 scores of the automated system while producing significant time savings for the human experimenter.

Existing deep-learning tools are mainly developed for the detection of solitary stomata in the commonly studied plant species (Aono et al. 2021; Gibbs and Burgess 2024). Examples include optical topometry (Xie et al. 2021) and the use of deep-learning (DL) technology to track stomata (Jayakody et al. 2017, 2021; Fetter et al. 2019; Casado-García et al. 2020; Bheemanahalli et al. 2021; Zhu et al. 2024). While the machine-learning strategy has proven successful for high-throughput phenotype analysis toward building correlations to genotypes (Millstead et al. 2020; Zhang et al. 2021), these existing tools have limited post-process data validation, review, and correction ability for the researchers. TESSERA not only provides multiple trait parameters but is also amenable to threshold filtering, thus allowing the researcher fast, fine-grained control over output data and can export it as common and easily accessible file formats.

The deep-learning tools developed in the past are primarily for solitary stomata analysis in the most commonly studied species of plants (Fetter et al. 2019; Gibbs and Burgess 2024; Wacker et al. 2025). We tested TESSERA against a diverse range of Begonia species (Fig. 1). TESSERA was deemed as reliable for analysis of different stomatal cluster patterns across a range of species and for analysis of trait segregation in mapping populations (Figs. 1–3, Figs. S2–S5). With TESSERA, parameter output data for patterning for both solitary and clustered stomata phenotypes are generated along with associated annotated image files for validation of the analysis.

Besides, TESSERA is amenable to the analysis of low resolution and automated slide scans; thus, its use benefits from wider tissue coverage and reduces experimenter time and financial investment. The human experts and TESSERA showed largely similar performance for data analysis, although surprisingly in some instances both the human experts and the machine did not perform as well as the human trainer (Fig. 3, Figs. S3–S5). It may be that TESSERA performance also depends on the average number of stomata per input image, with analysis of >100 stomata per image, as in CON being more reliable than input images with >150 stomata each as noted in PLE (Fig. 3a). We suggest that reliability in data analysis can differ between human users, attributed to human error that is not associated with experience, while the machine output would be consistent for input data as long as input image quality is maintained.

Some of the existing high-throughput stomata analysis tools require several pre-processing steps such as manual segmentation or training (Jayakody et al. 2021), including automatic segmentation of the images and user handling of object orientation to clarify model parameters. High-resolution or fluorescence images that required advanced imaging skills and equipment are also needed (Takaoka et al. 2020). TESSERA incorporates automated stomata localization through a refined YOLO v7 implementation, which may be adapted to newer versions of YOLO and related approaches such as Faster R-CNN. At this stage, we are already observing improved accuracy and an order-of-magnitude reduction in analysis time compared with the existing manual process. These observations suggest that future iterations of TESSERA will likely deliver further incremental gains through enhanced localization approaches while continuing to reduce the level of human involvement required. Human intervention and interactive manipulation, we propose, should be considered integral elements of machine-learning pipelines, rather than residual dependencies to be eliminated. Human–machine interactive deep-learning frameworks are a powerful mechanism for incorporating domain expertise into physiology and genetic studies, facilitating the discovery of novel factors governing plant physiology and underscoring the broader scientific value of expert-guided interaction.

### Stomatal patterning in *Begonia* underscores micromorphological variety and parameter correlations

Micromorphological variety underpins how plants thrive and adapt to a wide range of habitats and overcome environmental change. For instance, stomatal patterns, including guard cell size, shape, stomatal density, and arrangements on the leaf epidermis as solitary or in clusters, influence stomatal efficiency (Gray 2007). Past studies suggest that morphology traits, including stomatal density and size, are negatively correlated for solitary stomata (Hong et al. 2018; Haworth et al. 2023). For stomata occurring in clusters (Papanatsiou et al. 2017), the stomatal patterns are linked to the plant habitats and can impact how plants cope with environmental change (Liu et al. 2023). For instance, the genus *Begonia* occurs in diverse habitats (Moonlight et al. 2018), including regions with high temperatures that increase soil moisture evaporation and water loss through the stomatal pore (Sato et al. 2024). Although several *Begonia* species have retained ancestorial solitary stomata patterns, stomatal clusters have evolved in several *Begonias*, including species found across eastern South America, Caribbean, and western South America (Moonlight et al. 2018). We found solitary as well as cluster configurations of 2 and up to 12 stomata in our analysis of different *Begonia* species (Fig. 1). With over 2000 species growing in a huge variety of habitats, the genus represents a vast diversity in stomatal patterning (Hughes et al. 2015).

Reducing the size of the guard cells surrounding the stomatal pore or increasing the number of stomata within a cluster helps to adjust the plasticity of stomata (Franks and Beerling 2009). Without significant changes in the density of membrane transporters, the higher ratio of membrane surface area to guard cell volume increases solute flux per guard cell to achieve faster stomatal kinetics for improved stomatal efficiency (Jezek and Blatt 2017; Tsai et al. 2022). Past studies suggest that stomatal clustering also influences guard cell plasticity and can impede the speed with which stomata respond to environment change (Papanatsiou et al. 2017; Tsai et al. 2022). Indeed, in natural *Arabidopsis* clustering, mutants are impaired in stomatal movements, a feature arising from impaired solute exchange with neighboring epidermal cells (Dow et al. 2014; de Marcos et al. 2016). Thus, molecular factors that can integrate environment control into stomatal functions must act in stomatal development.

Our analysis of the *B. conchifolia* and *B. plebeja* parents and their backcrossed populations (CBC, PBC) suggested that stomatal clusters and density traits in *Begonia* are positively correlated, although this relationship is independent of the size of the cluster (Fig. 4). Thus, stomatal clustering promotes stomatal efficiency, as demonstrated before by (Papanatsiou et al. 2017). Stomatal spacing in cluster and the opening rates of the stomata are linked phenotypes (Tsai et al. 2022). Interestingly, we observed a negative correlation between size of the cluster and the spacing between adjacent stomata in a cluster (Fig. 4). In other words, stomata in larger clusters were more “crowded,” which could alter their plasticity (Papanatsiou et al. 2017; Tsai et al. 2022) and affect the speed with which stomata in these clusters respond to the environment. We therefore propose that cluster development regulates stomatal density and that 2 to 3 stomata clusters are likely a desirable trait for improving stomatal efficiency.

### Prediction of candidate genes related to stomatal development in *Begonias*

We have found 138 candidate genes in *Begonia* QTL analysis that have differential gene expression in *B. conchifolia* and *B. plebeja* species (Table 1, Table S4). These genes include transcription factor(s) regulated by phytohormones, including ABA that influences stomatal closure to prevent plant dehydration and is linked to influences on stomatal movements (Sanyal et al. 2017).

A significant number of the candidates we identified belong to gene/protein families that are already implicated in stomatal regulation, especially transcription factors from the *bHLH* family implicated in regulation stomatal development and in cold stress responses (MacAlister and Bergmann 2011). Transcription factor ICE1 (SCREAM1) homolog is the most well-studied transcription that forms heterodimers with SPEECHLESS (SPCH), MUTE, and FAMA mainly in *Arabidopsis* for the regulation of cell lineage differentiation in stomatal biogenesis (Gray 2007); SPCH initiates entry into the stomatal lineage; MUTE controls asymmetric divisions of stomatal precursor cells; and FAMA promotes guard cell differentiation (Pillitteri et al. 2007). *Begonia* SPCH protein has distinct structural domains as compared to its paralogs in *Arabidopsis*, and its expression drives stomatal clustering in *Arabidopsis* (Fig. 6b and c). Thus, we show that the study of *Begonia* stomatal patterning and genetics aided by TESSERA yields knowledge relevant to ecophysiology.

Several gene candidates encoding solute transporters and vesicle trafficking proteins are also identified. These could be new, yet uncharacterized factors influencing stomatal development and subjects for future studies. Indeed, solute transport and its coordination with vesicle traffic are known factors for dynamic control of guard cell volume and influence stomatal kinetics (Karnik et al. 2017; Baena et al. 2024; Yu et al. 2026). The family of anion channels SLAC1 is important for stomatal closure (Wang et al. 2012), the plasma membrane water channels aquaporin PIP proteins are implicated in guard cell turgor and abiotic stress responses (Baena et al. 2024), and the VAMP7 family of trafficking R-SNARE proteins are involved in vesicle trafficking and coordinate regulation of K^+^ channels in stomatal opening (Zhang et al. 2015, 2017). Thus, pathways that influence stomatal kinetics could be tied to the control of stomatal development. However, the knowledge of how trafficking mechanisms contribute to stomatal development is sparce. Past work showed that secretion of CO_2_-RESPONSE SECRETED PROTEASE (CRSP) that regulates stomatal development in high CO_2_ (Engineer et al. 2014) is mediated by a secretory SNARE (Xia et al. 2019). It may be that vesicle trafficking and ion transport pathways that coordinate stomatal movements likely integrate environment control into the developmental processes. Future studies should focus on these aspects.

In summary, we show that the combined use of deep-learning and human-machine interaction approaches is a reliable high-throughput strategy for building stomatal trait correlations. We demonstrate that the knowledge from *Begonia* gives unexpected new insights into plant development and it is important for both fundamental and applied research in the field.

## Materials and methods

### Plant lines and propagation

*Arabidopsis thaliana* ecotyp*e* Columbia Ø and *Begonia* species including *B. acetosella* (Acc: 19980065), *B. chiasmogyna* (Acc: 20170336), *B. chloroneura* (Acc: 19972555), *B. cleopatrae* (Acc: 19980289), *B. conchifolia* (Acc: 20042082), *B. corredorana* (Acc: 20071055), *B. foliosa* (Acc: 20180920), *B. glandulifera* (Acc: 20030497), *B. goegoensis* (Acc: 20070752), *B. henrilaportei* (Acc: 20160414), *B. involucrate* (Acc: 20070426), *B. nelumbiifolia* (Acc: 20100298), *B. odorata* (Acc: 20082086), *B. parviflora* (Acc: 20160148), *B. plebeja* (Acc: 20051406), *B. puspitae* (Acc: 20111539), *B. serratipetala* (Acc: 19681637), *B. subciliata* (Acc: 20141045), *B. sudjanae* (Acc: 20120808), and *B. ulmifolia* (Acc: 20030607) were used in this study.

Mapping populations used in the study were first produced (see Brennan et al. 2012) by crossing 2 phylogenetically closely related *Begonia* species *B. conchifolia* (CON), and *B. plebeja* (PLE) parent lines to generate the first hybrid generation plants (F1, Acc: CKB137, 8 plants). The F1 progeny was backcrossed with the original parent species to obtain the backcrossed populations; *B. conchifolia* backcross progeny (CBC, Acc: ARB, 44 plants) and *B. plebeja* backcross population (PBC, Acc: B08, 210 plants).

All *Begonia* plants were grown in the glasshouse at Royal Botanical Garden Edinburgh (RBGE), with day temperature of 28 °C and a night temperature of 20 °C and at 70% relative humidity (RH). For soil-grown *Arabidopsis*, seedlings were transplanted to 7-cm pots prior to propagation for 4 to 5 weeks in environment-controlled GEN1000 growth chambers (Conviron, Isleham, UK) using 16-h-light/8-h-dark, 22 °C/18 °C cycles under 150 μmol m^−2^ s^−1^ white light (total daily fluence of 4.2 mol m-2 PAR), and 55% RH.

### *Arabidopsis* transgenics preparation

*Beg*SPCH obtained by polymerase chain reaction (PCR) with gene specific primers (5′ ATGGGTGATATTTTGTCGG and 3′ GCAGAAGGTTTGCTGAATT) with cDNA from *B. plebeja* and *B. conchifolia* as template was cloned using Gateway GFP-tagged expression vector with *At*SPCH promotor (Lampard et al. 2008). *Arabidopsis* transgenic lines expressing *At*SPCH (At5G53210) and *Beg*SPCH were prepared as described before (Xia et al. 2019) by floral dipping (Clough and Bent 1998) of heterozygous *Arabidopsis spch-3^ht^* mutants (Lampard et al. 2008) with *Agrobacterium tumefaciens* GV3101 containing the expression vectors. Transformed plants (T2 generation) were obtained using kanamycin selection and leaf imprints were collected for stomatal parameter analysis from soil grown plants (as above).

### Leaf-epidermis imprints and image acquisition

There are variations in plant form in the mapping population due to the considerable differences between CON and PLE parents (Emelianova et al. 2021). Hence for consistency, fully expanded third or fourth leaves from the rhizome of 6- to 7-month-old fully grown plants prior to flowering (9 months) were chosen for sample collection. Epidermis imprints were obtained from each of the 4 fixed leaf regions on the left, right, tip, and petiole, which included regions of high stomatal density (Tang et al. 2002).

Prior to preparing imprints, the abaxial surface of leaves was cleaned using air spray and clear Sellotape to remove trichomes, dirt, and air bubbles. Leaf imprints of cleaned leaf surface were collected as described before (Wacker et al. 2025). Briefly, dental resin mix prepared by mixing catalyst and base (PRESIDENT light body kit, Coltene, Switzerland, #60019933) at a ratio of 0.6:0.4 was applied to a chosen region of the leaf and allowed to air dry (10 to 20 min). The dry imprint (negative) was carefully peeled and transferred to a glass slide, with the imprint facing up. A coat of nail hardener was applied and allowed to air-dry (30 to 45 min) before transferring the positive imprint to Sellotape (Sellotape Transparent Clear, Sellotape, UK, #1569594), which was mounted on a glass slide for imaging. Resin imprints were stored.

Images of *Begonia* leaf epidermis imprints were acquired using automated slide scanner (Motic Easy Scan Infinity, Motic, China) at 20× resolution using the Veterinary Diagnostic Services for Histology Research at the School of Biodiversity, One Health & Veterinary Medicine, University of Glasgow or an Axiovert 200 Inverted microscope (Zeiss, Germany), at 5× magnification. For additional parameter analysis, images were acquired using Leica M165 C (Leica, Germany). For data analysis, images of epidermis imprints were extracted using Aperio Image scope (v12.4.6) software at 10× magnification and 200 pixels per inch (ppi) resolution from at least 3 different randomly chosen positions each, spanning an area of A = 1.801213181 mm^2^. For manual analysis, image data was scored using and FIJI/ImageJ software (Schindelin et al. 2012).

### TESSERA machine learning and data processing

#### Machine learning and processing

TESSERA machine model was trained using a Dell 5,000 computer with Intel Xeon W-2145, 64GB RAM, and NVIDIA Titan RTX with 24GB RAM and 4,608 cores. The underlying machine learning approach for stomata localization was based on YOLO version 7 (Redmon et al. 2016; Joseph and Ali 2018), which is an established model for single-pass approach to object localization within images. We modified this approach to improve the sensitivity for stomata detection by adjusting the acceptable lower confidence threshold to 0.25. The resulting candidate regions were merged based on an overlap threshold of 0.5, and revised confidence was computed as the mean of the region confidence values weighted by the respective areas, motivated by weighted box fusion (Solovyev et al. 2021) with ensemble approaches to object localization (Vyas et al. 2022).

The approach contains 2 fully connected layers preceded by 24 convolution layers, the first 20 of which are pretrained on ImageNet (Deng et al. 2009) and the model is then further refined on annotated stomata images (see below). The refined localizations of the stomata are finally grouped into low, medium, and high confidence based on the weighted confidence score, thresholds at 0.25, 0.5, and 0.75, respectively, to generate preliminary annotations for images of the leaf epidermis imprints.

#### Image data and annotation

We used 11 images of *B. conchifolia* leaf imprints, size 934 × 1,716-pixels, that included 87 ± 11.5 stomata per image patched into 600 linearly spaced 224 × 224-pixel subregions, resulting in 6,600 images. Similarly, 8 images from *B. plebeja* leaf imprints were used, patched into 4,800 subregions, with 87.9 ± 19.2 stomata per image, each annotated by human experts.

#### Augmentation

Data augmentation included color space perturbations via HSV (hue, saturation, value) adjustments with ranges of ± 0.015, ± 0.7, and ± 0.4, respectively; spatial augmentations with translation of ± 0.2 and scale up to 0.9; horizontal flipping with likelihood 0.5; mosaic image composition for every image to increase object scale variation and context diversity during training; as well as MixUp blending and paste-in augmentation, each with a likelihood of 0.15.

#### Hyperparameters

The loss function consisted of weighted components for bounding box regression, classification, and objectness, with weights of 0.05, 0.3, and 0.7, respectively. The model was trained using stochastic gradient descent with initial learning rate of 0.01, decayed using a cosine learning rate scheduled to 0.001. Momentum was set to 0.937 and weight decayed to 0.0005. A 3-epoch warm-up was used along with intersection over union training threshold of 0.2 and anchor matching threshold of 4. Optimal transport assignment loss was used to improve label matching during training.

#### Human-machine interaction and data post-processing

The web-based human-computer interface of TESSERA displays the original analyzed image with annotations using model-derived bounding boxes superimposed. The interface also produces a gallery of thumbnail images corresponding to each detected stoma, accompanied by a unique identifier and the associated confidence score. Human users can refine the candidate set by adjusting the minimum acceptable confidence threshold, thereby filtering out low-confidence detections (true negatives). Users may also manually reinstate excluded candidates deemed to be valid stomata (“Mark as Stomata”, correcting false negatives) or remove high-confidence detections identified as incorrect (“Not Stomata”, correcting false positives). The curated outcomes are subsequently used to populate a confusion matrix for the selected threshold, enabling quantitative assessment of model performance. This step provides fine-grained human oversight, allowing users to inspect model predictions, rectify edge cases of misclassification, and improve the reliability of the final annotated dataset.

#### Density-driven cluster parameter analysis

Clustering of stomata was performed using Density-Based Spatial Clustering of Applications with Noise (DBSCAN) (Ester et al. 1996), which groups points according to local density rather than imposing geometric assumptions on cluster shape. This method was selected over alternatives such as k-means clustering (MacQueen 1967) due to its computational efficiency, robustness to noise arising from imperfect localization, and ability to operate without a predefined number of clusters. This approach was considered as desirable, since the number of cluster and individual cluster density are unknown variables for each source image. DBSCAN iteratively constructs clusters by aggregating stomata whose pairwise distances fall within a specified neighborhood, continuing this process until all points are assigned to a cluster or designated as noise. The minimum cluster size was set to 2 stomata. The neighborhood radius (epsilon) was computed dynamically for each scan by estimating the mean bounding-box size of the filtered candidates and scaling this value by a factor of 2. This adaptive strategy ensures scale invariance across scans with differing image resolutions and stomatal sizes.

The pipeline outputs an annotated image file, in which each stoma is shown as connected to its corresponding cluster unless it is not part of a cluster. Supplementary data, including cluster assignments, inter-stomatal distances, and summary statistics, are exported as.csv files, to support downstream quantitative analysis.

### Test of TESSERA-based analysis

The performance of TESSERA in stomatal analysis was determined using 11 phenotypic trait measurements (cf. Table S1), comparing against human Trainer as gold standard and with 2 additional human experts (researchers).

#### F1-score calculations

F1 score was calculated as the balance between the harmonic means between precision and recall (Powers 2011; Wacker et al. 2025), with precision determining efficiency of the machine in recognizing artefacts that are truly stomata, while the recall indicating machine sensitivity for detecting stomata as artefacts in the image. The machine-annotated scores for artefact detection and classification in each image file following analysis of at fixed threshold t = 0.29 were classed as True True (TT) for artefacts detected and correctly classified as stoma, True False (TF) for non-stomata artefacts detected that were misclassified as stoma, False True (FT) for artefacts with stomata that were misclassified as non-stomata by TESSERA, and False False (FF) where TESSERA correctly excluded an artefact from both detection and classification as stoma. The human Trainer analyzed the annotated image file outputs from TESSERA and scored any unannotated stomata artefacts (U) in the image that the machine failed to detect.


$$Precision=\frac{(TT)\;}{\left(TT\;+\;TF\right)}$$

$$Recall=\frac{(TT)\;}{\left(TT+FT+U\right)}\;$$

$$F1=2\times \frac{\left(Precision\times Recall\right)}{\left(Precision+Recall\right)}$$

F1 score >0.9 was considered as excellent, 0.8< F1 < 0.9 as good, and F1 < 0.5 was considered as poor for TESSERA reliability (Powers 2011).

#### Concordance correlation coefficient calculation

To determine the agreement between the machine and human researchers, concordance correlation coefficient (CCC) (Lin 1989) and ordinary least squares (OLS) regression analysis were employed. CCC values of ≥ 0.8 are used as an indication for an excellent fitting between 2 methods and ≤ 0.2 as weak correlations. Statistical tests were carried out to determine significance (see below).

### Stomatal trait analysis

Following image analysis, parameter data were calculated relative to unit image area (A).


$$Stomatal\;cluster\;density=\frac{\left(number\;of\;stomatal\;clusters\right)}{\left(A\right)}$$

$$Stomata\;per\;cluster=\frac{(number\;of\;stomata\;in\;all\;clusters\;in\;each\;image)\;}{\left(number\;of\;clusters\;in\;each\;image\right)}$$

Additional phenotypic traits included in QTL analysis were as described by (Ali 2013) and included cluster length, cluster width, and spacing between clusters.

### Principal component analysis (PCA)

Principal component analysis PCA was performed in R (https://search.r-project.org/R/refmans/stats/html/prcomp.html) and visualized by ggbiplot package (https://cran.r-project.org/web/packages/ggbiplot/index.html). Variable extractions from PCA included contribution of variables generated using factoextra packages (https://cran.r-project.org/web/packages/factoextra/index.html).

### Pearson correlations and heat map

Pearson correlation analysis was carried out using Python by pandas.DataFrame.corr package. Correlation matrix of 11 stomatal traits was represented using seaborn.clustermap (https://seaborn.pydata.org/generated/seaborn.clustermap.html).

Mean of traits for each species analysed were presented as heatmap using seaborn.clustermap (https://seaborn.pydata.org/generated/seaborn.clustermap.html). Ward linkage method was used to calculate the clusters. Euclidean metric was applied to measure the distance of data.

### Genetic mapping

Genomic DNA was extracted from 210 plants of the *Begonia* PBC mapping populations. Genome skimming for each plant was performed with Illumina PE150 with 5× coverage [cf. (Fan 2023)]. Reads were mapped to a genome assembly for *B. conchifolia* (Campos-Dominguez et al. 2025) using Bowtie2 (Langmead and Salzberg 2012), and SNPs called using Freebayes (Garrison and Marth 2012) and GATK (Langmead et al. 2012; Garrison et al. 2012; Auwera and O'Connor 2020). The R package OneMap was used to filter and generated the genetic map (https://cran.r-project.org/web/packages/onemap/) on the UK Crop Diversity server (Percival-Alwyn et al. 2025) to obtain a final map of 14 linkage groups with 2,389 genetic markers.

### QTL analysis

QTL analysis was performed using the qtl2 packages from R (https://cran.r-project.org/web/packages/qtl2/). A total of 187 plant individuals from the *Begonia* PBC mapping populations was used. Phenotypic data comprised of 16 cluster parameters (see above), including the 11 stomatal trait data scored using TESSERA, PC1 and PC2 individual plant values extracted using PCA analysis as covariance control, and manually scored data for 3 additional traits. To reduce the errors of trait variances through large surface during leaf development stage, trait scores from additional leaf regions from the leaf tip, right, left and petiole were chosen for stomatal density, number of solitary stomata, number of clusters and average number of stomata per cluster. Single QTL model was used to perform a genome scan on the matrix of cluster phenotypic traits. Phenotypic traits of leaf including light saturated net photosynthesis (A_max_), leaf area (Area), specific leaf area (SLA), depth of mesophyll, depth of palisade mesophyll, and depth of spongy mesophyll previously generated by (Ali 2013) were used as additive co-variants in single QTL model (https://search.r-project.org/CRAN/refmans/qtl/html/scanone.html). Traits with LOD score > 3.0 were identified as significant. Percentage of explanation of variances (%var) by each QTL was calculated as before (Broman et al. 2003) where n is the plant sample size (187 for both genotype and phenotype).


$$\text{\%}var=1-10^{\frac{-2}{n}LOD}$$

The *B. conchifolia* scaffolds under the confidence interval of each QTL were extracted and annotated genes examined for candidate genes.

#### Additive covariant analysis in QTL mapping

Phenotypic traits of leaf including light saturated net photosynthesis (A_max_), leaf area (Area), specific leaf area (SLA), depth of mesophyll, depth of palisade mesophyll, and depth of spongy mesophyll previously generated by (Ali 2013) were used as additive co-variants in single QTL model (https://search.r-project.org/CRAN/refmans/qtl/html/scanone.html).

#### Short listing of QTL candidates by differential gene expression (DEGs) analysis

RNA-Seq data was previously generated using *B. conchifolia* mature leaf and vegetative bud tissue (Emelianova et al. 2021). Raw reads were mapped to the gene space from the reference genome of *B. conchifolia* (Campos-Dominguez et al. 2025). The transcript abundance was obtained using SALMON (Patro et al. 2017) and quantified using R as described by (Emelianova et al. 2021). DGE analysis was conducted as described by (Guo et al. 2021) using 3D RNA-Seq tool for analysis of RNA-seq data (https://3drnaseq.hutton.ac.uk/app_direct/3DRNAseq/). The weighted trimmed mean of M-values (TMM) method was applied for normalization of data. The QTL candidates were found using sed, unix and grep tool in Python by linking the *B. conchifolia* scaffold of each QTL to the genetic markers presenting differential gene expression between the 2 different tissues among *B. conchifolia* and *B. plebeja*. Protein sequences of candidates were extracted. The identity of each protein confirmed using protein BLASTp on the NCBI (https://blast.ncbi.nlm.nih.gov/Blast.cgi).

### Statistical analysis and graph representation

Anderson-Darling test, D’Agostino and Pearson test, Shapiro-Wilk test, and Kolmogorov-Smirnov tests were applied to determine normalized distributions of the datasets. Significant differences were determined using ANOVA for multiple comparisons and with parametric Brown–Forsythe or Welch *t*-test or non-parametric Kruskal-Wallis and Mann-Whitney tests. Statistical differences between means were calculated using GraphPad Prism (v8.4.3), with *P* values <0.05 considered as significantly different.

PCA plots and LOD plots for QTL analysis were generated using R programming language (v4.3.1). Python and R programming analysis was performed on Jupyter notebook (v6.5.2) on Anaconda platform (v2.6.2).

## Supplementary Material

kiag496_Supplementary_Data

## Data Availability

The data that supports the findings of this study are available in the Supplementary material of this article (cf. Tables S1–S4). Vectors are available at https://plantscienceglasgow.org. TESSERA is available with Bryan Williams (b.williams6@lancaster.ac.uk) and Abhijit Karnik (a.karnik@lancaster.ac.uk).
