# Supplementary material for: Genetic markers of stomatal cluster development in Begoniaceae revealed through trait analysis assisted by interactive deep-learning
Source: Plant Physiol. 2026 Jul 13;201(3):kiag496. doi: 10.1093/plphys/kiag496 (PMC13418361; doi:10.1093/plphys/kiag496)
Supplement: kiag496_Supplementary_Data [file kiag496_supplementary_data.zip › PLPHYS-2026-0421_R1_ TESSERA Supplemental Figures.docx]

**Supplemental Figures.**


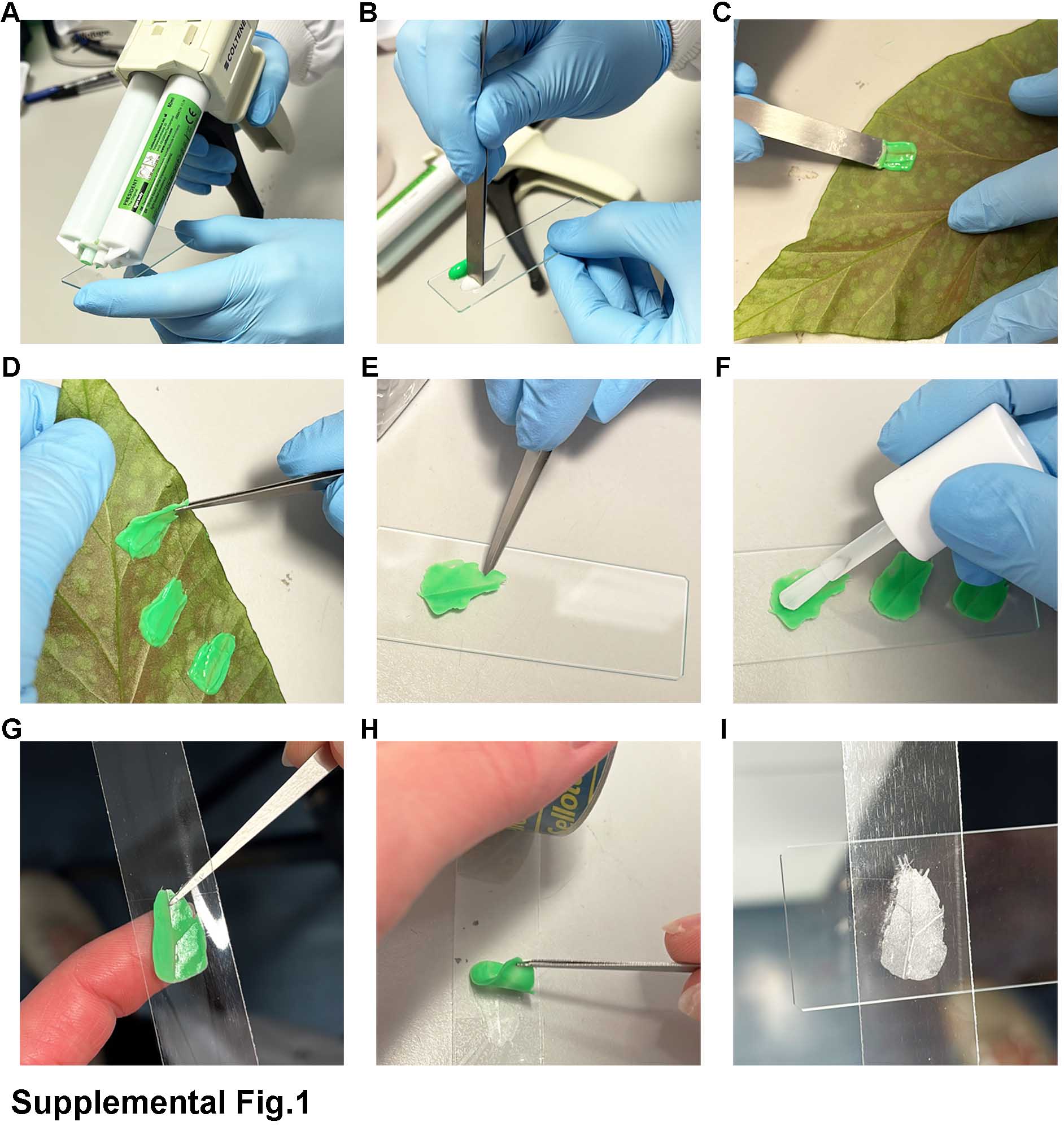


**Supplemental Fig.1 Leaf epidermis imprint preparation.** Workflow is depicted using photos of key steps in sequential order. Panels show (**A-B**) mixing of components for of resin from dispenser, (**C**) application of resin mixture on the abaxial surface of a leaf, (**D**) peeling of dried resin imprint from the leaf after approx. 10-20 min), (**E-F**) applying nail varnish on the resin imprint to create positive imprint, (**G-H)** transfer of positive imprint to Sellotape, (I) Sellotape with imprint impression transferred to a glass slide prior to imaging (also see Methods).


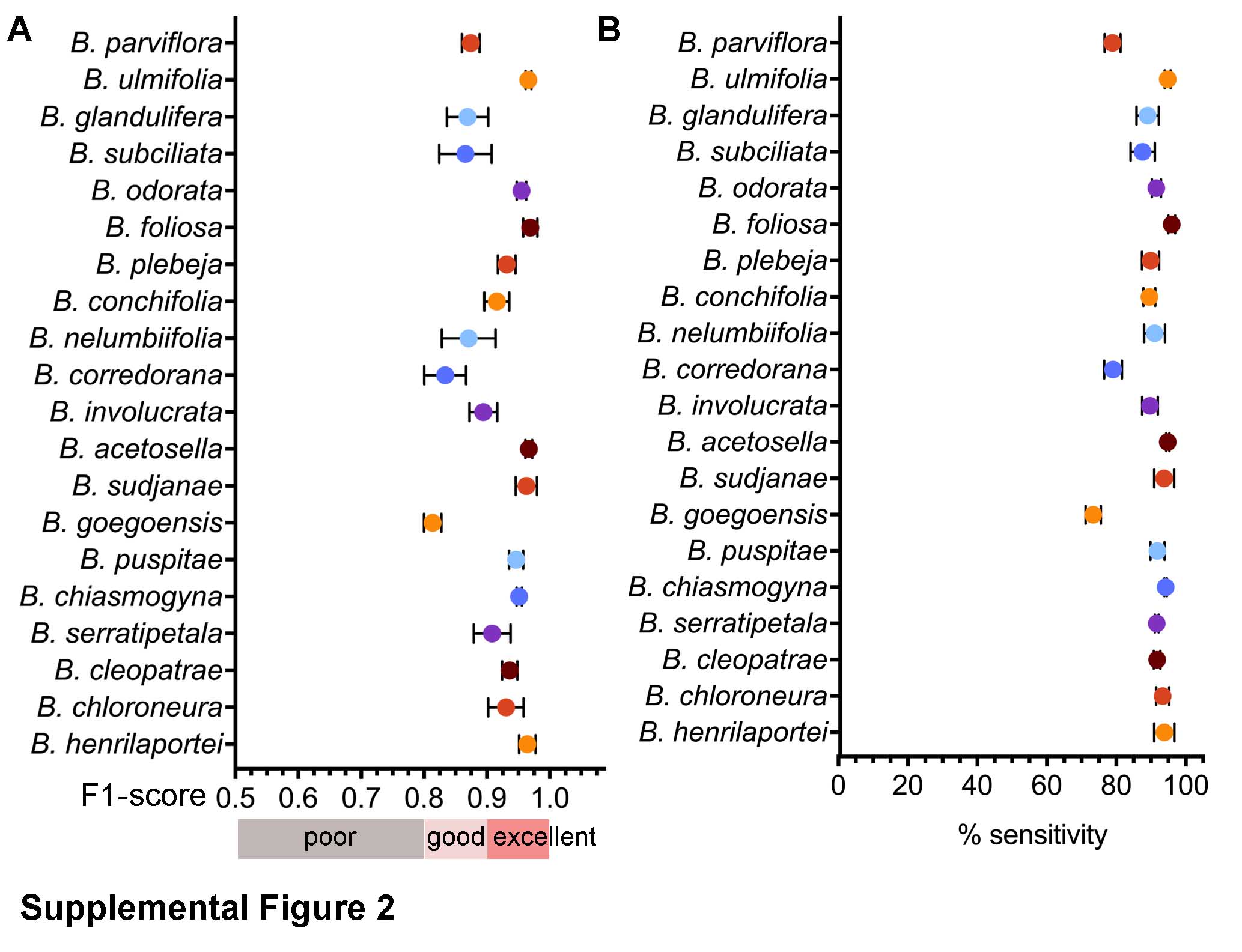


**Supplemental Figure 2. Test of TESSERA reliability.** Plots are mean ±SE for (**A**) F1-scores and (**B)** %sensitivity depicting TESSERA reliability in stomata detection in leaf imprint images (n=3 each). % sensitivity was derived as percentile of recall (see Methods) for stomatal density parameter and comparing outputs from TESSERA with reference to that of the gold standard human trainer. Analysis of 20 *Begonia* species (indicated) was included. F1 scores (see Methods) for machine performance were interpreted as very good (F1 >0.9), good (F1 =0.8-0.9), moderate ( F1=0.5 - 0.8) and poor (F1 < 0.5).

**
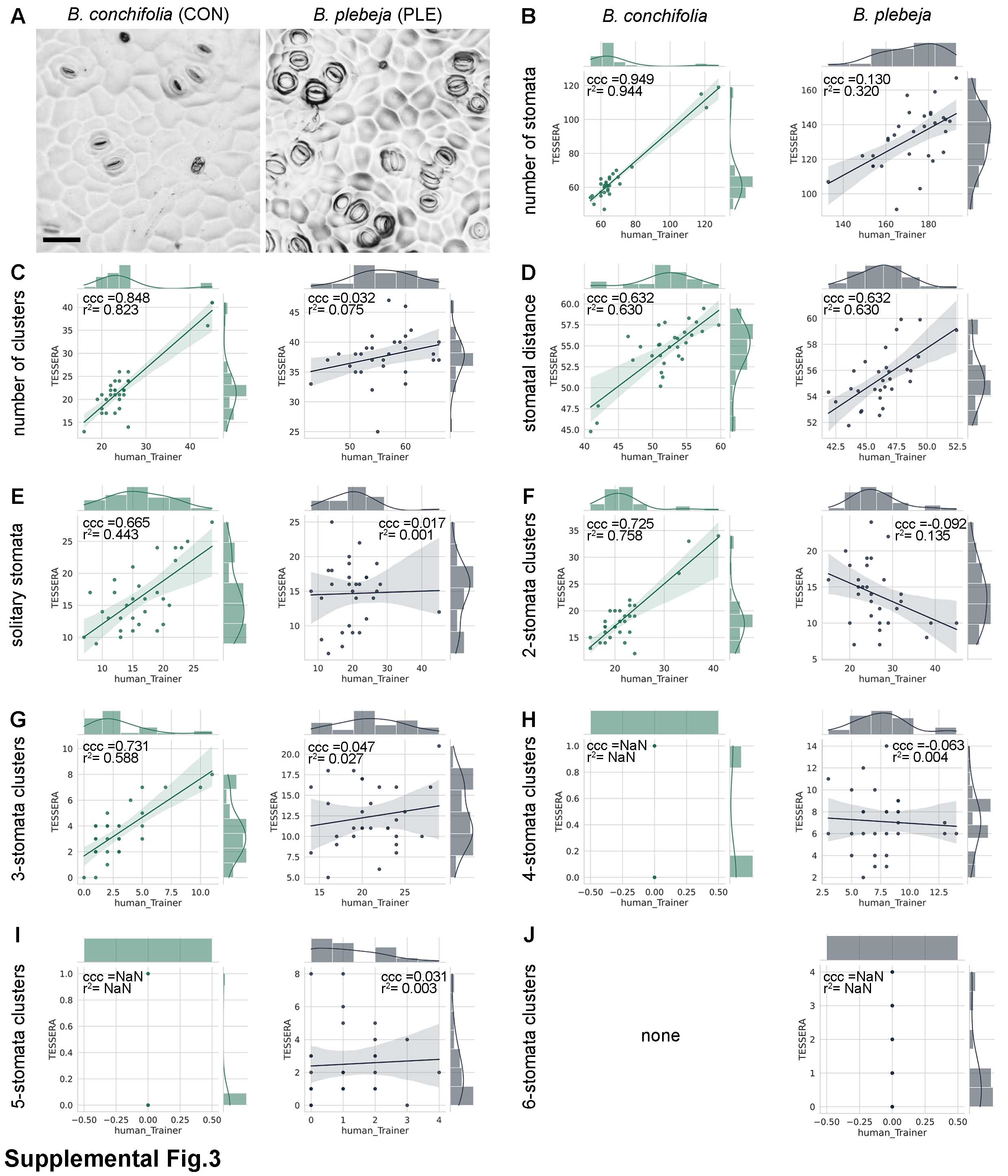
**

**Supplemental Figure 3. Evaluation of TESSERA learning**. (**A**) Representative images of imprints from *B. conchifolia* (CON) and *B. plebeja* (PLE) are shown. Scale bar =100 μm. (**B-J**) Regression plots following analysis of Concordance Corellation Coefficient (CCC) and OLS regression are shown with agreement level (ccc) and predictive power (r2) of TESSERA compared against human expert for number of stomata (**B**), number of clusters (**C**), distance between adjacent stomata in a cluster (**D**), number of solitary stomata (**E**), number of 2-stomata clusters (**F**), number of 3-stomata clusters (**G**), number of 4-stomata clusters (**H**), number of 5-stomata clusters (**I**) and number of 6-stomata clusters (**J**). n =30.

**
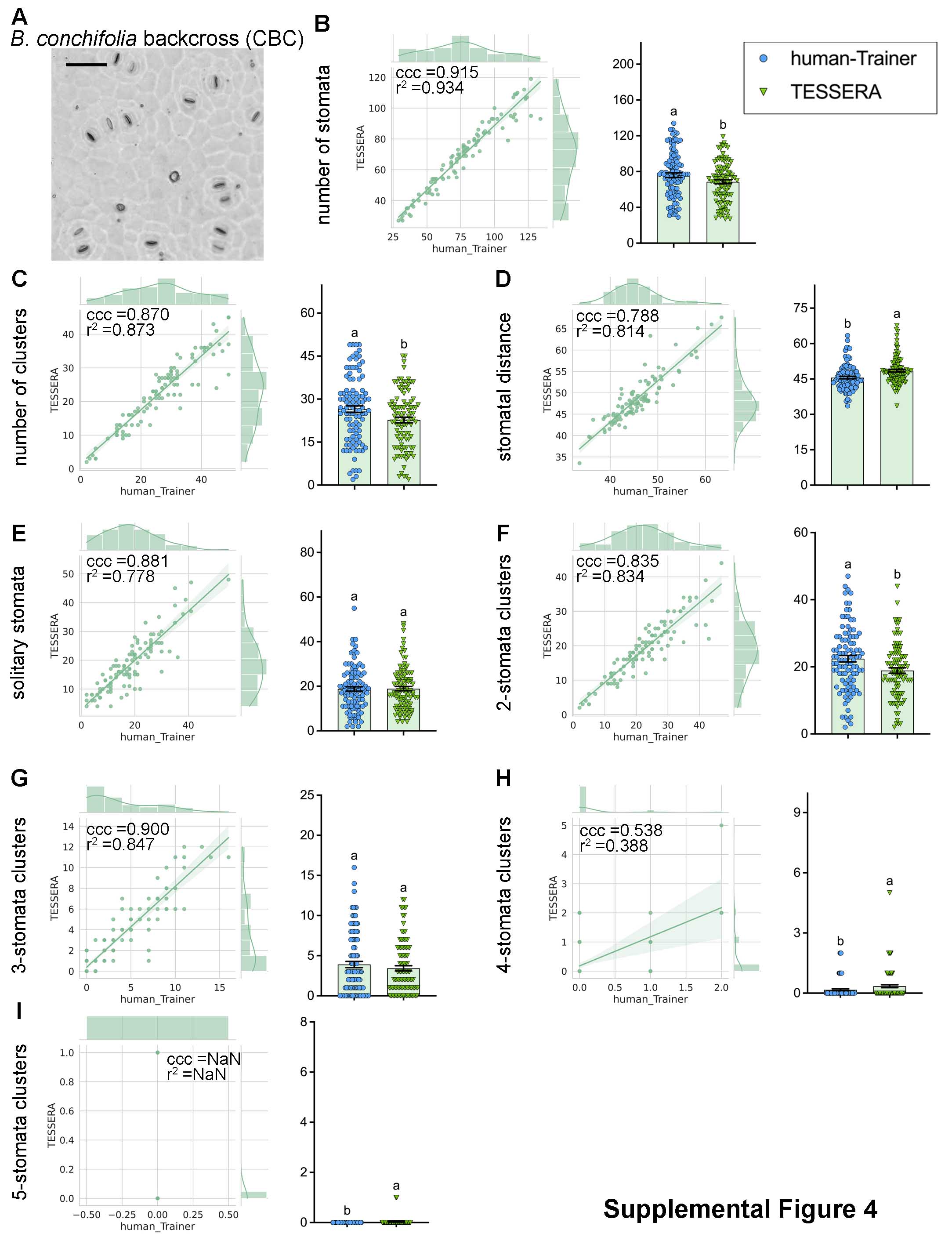
**

**Supplemental Fig.4 Evaluation of TESSERA performance using *B. conchifolia* backcrossed progeny (CBC).** (**A**) Representative image of imprints from ***CBC*** is shown. Scale bar =100 μm. (**B-J**) Regression plots (left panel) following analysis of Concordance Corellation Coefficient (CCC) and OLS regression are shown with agreement level (ccc) and predictive power (r2) and mean ±SE (right panel) of data for TESSERA (inverted triangle) compared against human Trainer (circle). Traits measured are number of stomata (**B**), number of clusters (**C**), distance between adjacent stomata in a cluster (**D**), number of solitary stomata (**E**), number of 2-stomata clusters (**F**), number of 3-stomata clusters (**G**), number of 4-stomata clusters (**H**) and number of 5-stomata clusters (**I**). n=100. Statistical significance depicted using letters was determined using Mann-Whitney and Welch’s t-test, (p < 0.05).

**
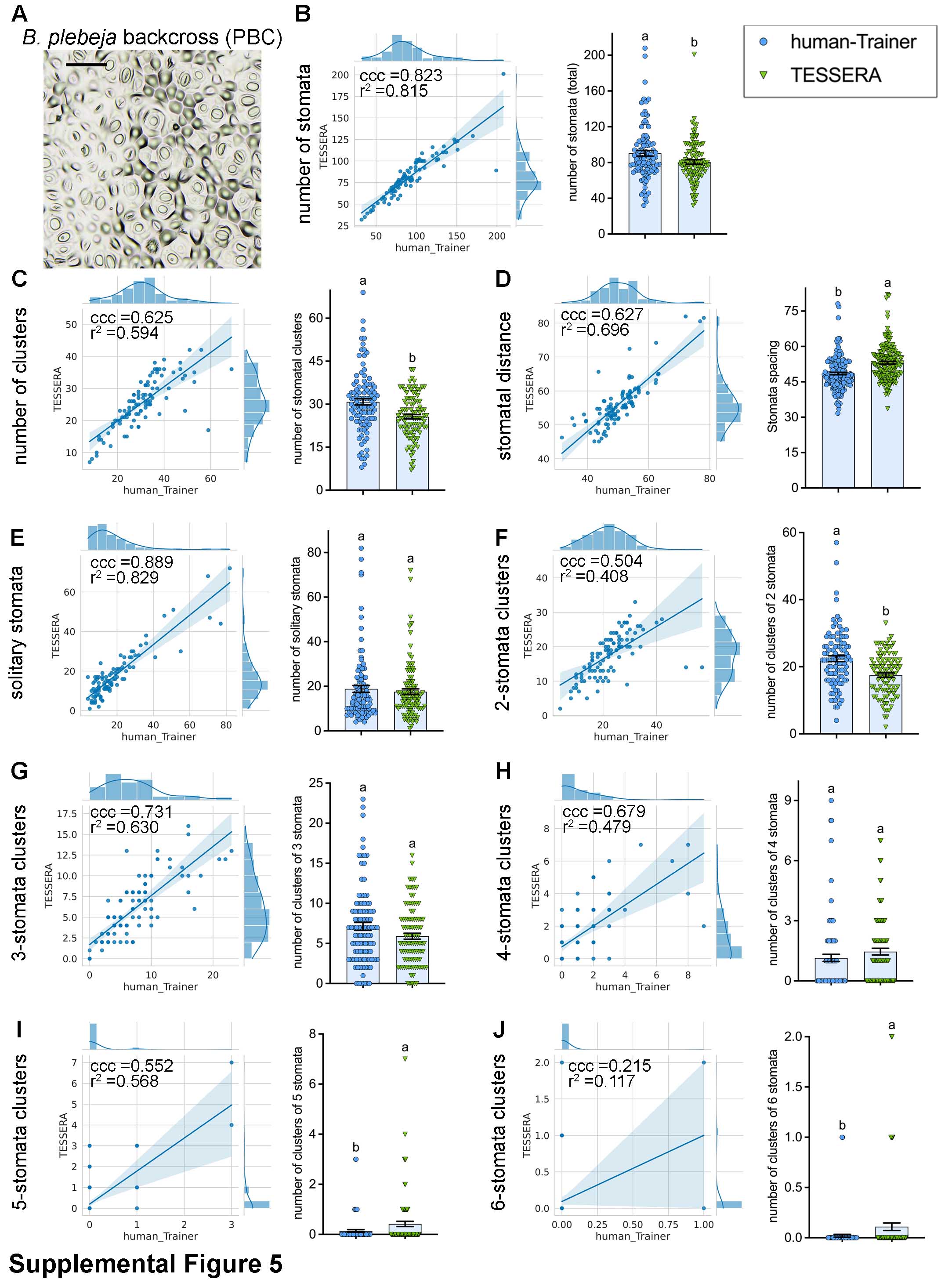
**

**Supplemental Fig.5 Evaluation of TESSERA performance using *B. plebeja* backcrossed progeny (PBC).** (**A**) Representative image of imprints from ***CBC*** is shown. Scale bar =100 μm. (**B-J**) Regression plots (left panel) following analysis of Concordance Corellation Coefficient (CCC) and OLS regression are shown with agreement level (ccc) and predictive power (r2) and mean ±SE (right panel) of data for TESSERA (inverted triangle) compared against human Trainer (circle). Traits measured are number of stomata (**B**), number of clusters (**C**), distance between adjacent stomata in a cluster (**D**), number of solitary stomata (**E**), number of 2-stomata clusters (**F**), number of 3-stomata clusters (**G**), number of 4-stomata clusters (**H**), number of 5-stomata clusters (**I**) and number of 6-stomata clusters (**J**). n=100. Statistical significance is depicted using letters and was determined using Mann-Whitney and Welch’s t-test, (p < 0.05).

**List of Supplemental Tables.**

Supplemental Table 1. Phenotypic traits for QTL analysis

Supplemental table 2. PCA components and variables.

Supplemental table 3. %Contribution of variables in PCA.

Supplemental table 4. Begonia candidates identified in QTL for stomatal traits.

Supplemental table 5. Significant QTLs for stomatal traits with co-variants for leaf anatomy and physiology traits.

Supplemental table 6. Begonia candidates identified in stomatal traits wiith leaf anatomy co-variant.
